# Absence of systemic toxicity changes following intramuscular administration of novel pSG2.HIVconsv DNA, ChAdV63.HIVconsv and MVA.HIVconsv vaccines to BALB/c mice^☆^

**DOI:** 10.1016/j.vaccine.2013.06.068

**Published:** 2013-11-12

**Authors:** Beatrice Ondondo, Caroline Brennan, Alfredo Nicosia, Steven J. Crome, Tomáš Hanke

**Affiliations:** a The Jenner Institute, University of Oxford, Old Road Campus Research Building, Roosevelt Drive, Oxford OX3 7DQ, United Kingdom; b Huntington Life Sciences, Huntington PE28 4HS, United Kingdom; c Okairòs, Rome, Italy; d CEINGE, Naples, Italy

**Keywords:** Pre-clinical toxicity, Modified vaccinia virus Ankara (MVA), Plasmid DNA, Chimpanzee adenovirus, HIV-1 vaccines, Conserved regions of HIV-1, MVA, modified vaccinia virus Ankara, Hct, haematocrit, Hb, haemoglobin, RBC, erythrocyte, MCHC, mean cell haemoglobin concentration, MCV, mean cell volume, WBC, total white cell count, LUC, large unstained cells, Plt, platelet, ALP, alkaline phosphatase, ALT, alanine aminotransferase, AST, aspartate aminotransferase, gGT, gamma-glutamyl transpeptidase

## Abstract

**Background:**

The systemic toxicity of three candidate HIV-1 vaccines plasmid pSG2.HIVconsv DNA (D), ChAdV63.HIVconsv (C) and MVA.HIVconsv (M) expressing chimeric immunogen derived from the most conserved regions of the HIV-1 proteome was evaluated in two repeat-dose studies in the male and female BALB/c mice.

**Methods:**

In study UNO011, mice received three doses of 2 × 10^7^ plaque-forming units of MVA.HIVconsv vaccine (MMM). In study UNO012, mice received 3 doses of 50 μg of pSG2.HIVconsv DNA followed by a single dose of 5.95 × 10^9^ virus particles of ChAdV63.HIVconsv vaccine (DDDC). Similarly constituted control groups received the vehicle alone (phosphate buffered saline) at the same volume-dose. All vaccines were administered by intramuscular needle injection into the right hind limb at 14-day intervals and animals were sacrificed 7 days after the last dose. Assessment of local and systemic toxicity was made. Induction of HIV-1-specific responses was confirmed. Parameters assessed included clinical condition, body weight, food consumption, ophthalmoscopy, haematology, blood chemistry, organ weight and macroscopic and microscopic pathology.

**Results:**

In both studies, treatment with the candidate vaccines elicited strong HIV-1-specific T-cell responses. The vaccine treatment was well-tolerated without any adverse systemic toxicological changes. The local toxicity findings observed in these studies were consistent with the predicted response to a vaccine/substance administration by intramuscular injection.

**Conclusions:**

The three novel anti-HIV-1 vaccines were well tolerated when administered by intramuscular injection to BALB/c mice. These results supported an application for authorisation by the Medicines and Healthcare Products Regulatory Agency of the UK to test these vaccines for the first time in phase I clinical trials in healthy both uninfected subjects and HIV-1-infected patients stable on antiretroviral treatment.

## Highlights

• Three novel vaccines were tested in 2 GLP toxicity studies in BALB/c mice.
• Vaccines were vectored by DNA, simian adenovirus and modified vaccinia virus Ankara.
• All 3 vaccines expressed the same conserved regions of HIV-1.
• Intramuscular administration had no systemic toxicity.
• All changes were consistent with i.m. delivery and immune response induction.

## 1 Introduction

HIV-1 infection/AIDS continue to be a major health challenge worldwide, but with more devastating consequences in low-resource settings such as sub-Saharan Africa and South East Asia, where the majority of HIV-1-infected people live. A vaccine is urgently needed, which would on its own or in conjunction with highly active anti-retroviral treatment prevent new infections, lower the risk of transmission and/or prevent progression to AIDS. However, efforts to develop such vaccines are hampered by several factors including the enormous HIV-1 diversity, and lack of simple and consistent correlates of immune protection, which would provide reliable surrogates of vaccine efficacy [1].

HIV-1 vaccines derived from conserved regions of the proteome could provide an answer to the problem of HIV-1 variability and escape from immunological control [2]. HIVconsv, a novel T cell immunogen designed specifically to overcome the antigenic variation of HIV-1, is a chimeric protein assembled from the 14 most highly conserved domains among the HIV-1 clade A, B, C and D proteomes [3]. Several heterologous vaccine modalities and their combinations were explored to maximise the induction of HIV-1-specific T-cell responses [4–8]. Here, three of these vaccines, namely pSG2.HIVconsv, ChAdV63.HIVconsv and MVA.HIVconsv vectored by respective plasmid DNA, non-replicating adenovirus of chimpanzee origin and non-replicating modified vaccinia virus Ankara, were evaluated in pre-clinical repeat dose studies in BALB/c mice to assess possible toxic effects associated with intramuscular administration prior to their use in humans.

## 2 Materials and methods

### 2.1 Vaccine production and formulation

The pSG2.HIVconsv DNA vaccine [8] was produced by the Clinical Biotechnology Centre (CBC), Bristol Institute for Transfusion Science, University of Bristol, UK by culture in *E. coli* strain XL-1 Blue. The pSG2.HIVconsv DNA vaccine (Batch no. Pilot 22/05/09) was formulated in phosphate buffered saline (pH 7.4) at 4.0 mg/ml and stored frozen at below −70 °C until use. The ChAdV63.HIVconsv vaccine [8] was produced at the Clinical Biomanufacturing Facility (CBF) of University of Oxford, UK, by suspension culture in HEK293 cells. The purified virus was diluted with formulation buffer to 1.35 × 10^11^ virus particles (vp)/ml and stored frozen at below −70 °C. The MVA.HIVconsv vaccine [8] was produced by IDT Biologika GmbH, Germany, by culture in chick embryo fibroblasts (CEF) prepared from Specific Pathogen Free embryonated hens’ eggs. MVA.HIVconsv was diluted in a formulation buffer to 5.5 × 10^8^ plaque-forming units (pfu)/ml and stored frozen below −70 °C. All cell culture, purification and aseptic processing steps in the manufacture of these vaccines were carried out according to the requirements of cGMP and using the GMP manufacturing process.

### 2.2 Animals

The mouse was chosen as the test species because of its acceptance as a predictor of toxic changes in man and the requirement for a rodent species by regulatory agencies. The BALB/c strain was used because it allows testing for vaccine potency. A total of 80 BALB/c mice (40 females and 40 males, 10 animals per study group) aged 6–7 weeks obtained from Charles River (UK) Ltd. were used in the study. The mice were within a weight range of 3 g for each sex.

### 2.3 Vaccine administration

The vaccines were administered as two separate studies. In UNO0012, three doses of 50 μg pSG2.HIVconsv (D) were given at 14-day intervals followed 14 days later by a single dose of 5.95 × 10^9^ vp of ChAdV63.HIVconsv (C) (regimen DDDC, Group 2). In UNO0011 MVA.HIVconsv (M) was administered on 3 occasions at 14-day intervals (regimen MMM, Group 4) at 2 × 10^7^ pfu per dose. Mice were killed 7 days after the last dose. Both studies utilised a control group which received vehicle (phosphate buffered saline (Groups 1 and 3)) at the same volume dose as the treated group on the same dosing occasions. Both studies were carried out in accordance with the principles of Good Laboratory Practice (GLP). The study groups, doses and schedule of administration are summarised in Table 1. All doses were given by intramuscular injection into the right hind limb.

### 2.4 Parameters assessed

The following parameters were examined as evaluation for toxicity. *Mortality and clinical condition* – animals were removed from the cage and observed at least twice daily during the study; on days of dosing detailed observations were recorded at more frequent intervals. Any deviation from normal in respect of nature and severity, date and time of onset, duration and progression was recorded. Local reactions were monitored and recorded by daily observations of the injection site. *Body weight* – the weight of each mouse was recorded one week before treatment commenced, on the day that treatment commenced (Day 1), twice weekly thereafter and before necropsy. *Food consumption* – the amount of food supplied to each cage and that leftover including estimated spillage was recorded for the week before treatment and each week throughout the study. *Ophthalmoscopy* – the eyes of all animals were examined by means of a binocular indirect ophthalmoscope, once before treatment commenced and one day after the last dose. Prior to each examination, the pupils were dilated using a tropicamide ophthalmic solution (Mydriacyl). The adnexae, conjunctiva, cornea, sclera, anterior chamber, iris (pupil dilated), lens, vitreous and fundus were examined. *Haematology (peripheral blood)* – at termination, animals were held under light general anaesthesia and blood samples (nominally 0.3 ml) were drawn into tubes containing EDTA anticoagulant and examined for the following characteristics using a Bayer Advia 120 haematology analyser: haematocrit (Hct), haemoglobin concentration (Hb), erythrocyte count (RBC), reticulocyte count, mean cell haemoglobin (MCH), mean cell haemoglobin concentration (MCHC), mean cell volume (MCV), total white cell count (WBC), differential WBC count and platelet count. *Blood chemistry* – at termination, further blood samples (nominally 0.6 ml) were collected into lithium heparin anticoagulant, centrifuge and the plasma analysed for the following blood chemistry parameters: alkaline phosphatase (ALP), alanine aminotransferase (ALT), aspartate aminotransferase (AST), urea, creatinine, gamma-glutamyl transpeptidase (gGT), total bilirubin, glucose, total cholesterol, triglycerides, sodium, potassium, chloride, calcium, inorganic phosphorus and total protein. *Pathology* – a full macroscopic and microscopic examination of a wide range of tissues was performed with a selection of organs weighed (Table 2). *Evaluation of vaccine potency and immunogenicity* – immunogenicity of the three HIVconsv vaccines was evaluated by testing pooled splenocytes for HIV-1-specific CD8^+^ T-cell responses by interferon (IFN)-γ ELISPOT assay or intracellular cytokine staining (ICS) using peptide H added to the C terminus of the HIVconsv protein [3]. Antibody responses to the vectors or insert were not measured.

### 2.5 Statistical analysis

All statistical analyses were carried out separately for males and females. Body weight was analysed using gains over appropriate study periods. Organ weights were analysed as absolute and adjusted for terminal body weight where appropriate. The following sequence of statistical tests was used for body weight, organ weight and clinical pathology data: A parametric analysis was performed if Bartlett's test for variance homogeneity was not significant at the 1% level. Groups were compared using *t*-tests. A non-parametric analysis was performed if Bartlett's test was still significant at the 1% level following both logarithmic and square-root transformations. Groups were compared using Wilcoxon rank sum tests. For clinical pathology data, if 75% of the data (across all groups) were the same value, Fisher's Exact tests were performed. Treatment groups were compared using pairwise comparisons of each dose group against the control. For organ weight data, analysis of covariance was performed using terminal body weight as covariate. The treatment comparisons were made on adjusted group means in order to allow for differences in body weight, which might influence the organ weights. Significant differences between control and treated groups were expressed at the 5% (*p* < 0.05) or 1% (*p* < 0.01) level.

## 3 Results

### 3.1 Vaccine take

**Fig. 1:**
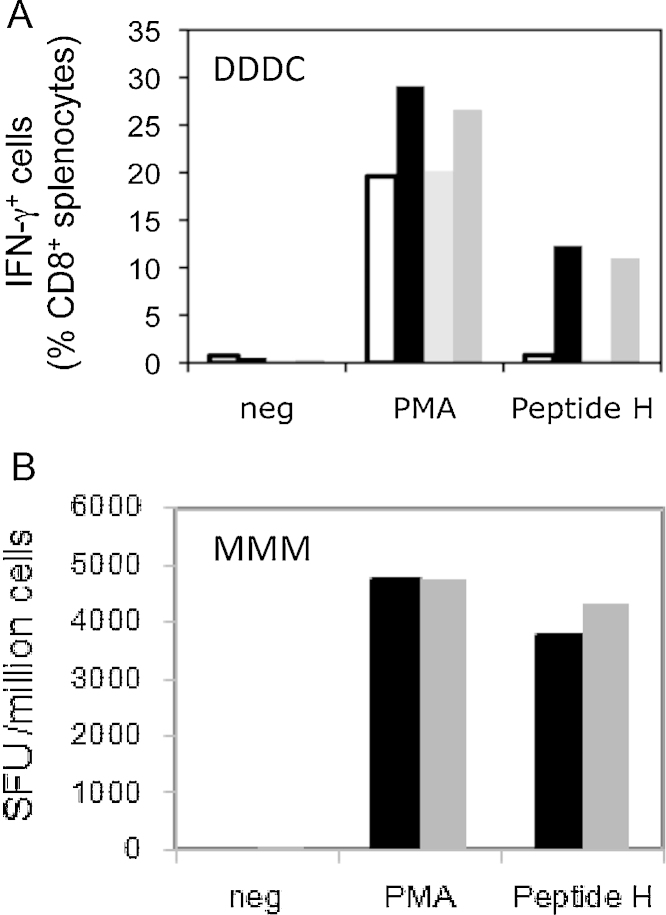
Induction of T-cell responses by the pSG2.HIVconsv DNA, ChAdV63.HIVconsv and MVA.HIVconsv vaccines. (A) Groups of 10 male and 10 female mice received 3 doses of 50 μg of pSG2.HIVconsv DNA followed by a single dose of 5 × 10^9^ vp of ChAdV63.HIVconsv at 2-week intervals. A similar (control) group received 4 doses of PBS. One week after the last dose, the mice were sacrificed and the splenocytes pooled for each group tested for IFN-γ production by intracellular cytokine staining. Neg – stimulation with no peptide (negative control); PMA – stimulation with PMA (positive control); peptide H – stimulation with H peptide present in HIVconsv immunogen; control males (white), control females (light grey), vaccinated males (black) and vaccinated females (dark grey). (B) Groups of 10 male and 10 female mice received 3 doses of 2 × 10^7^ pfu of MVA.HIVconsv at 2-week intervals. One week after the last dose, the mice were sacrificed and the splenocytes tested for IFN-γ production by ELISPOT assay. Neg – stimulation with no peptide (negative control); PMA – stimulation with PMA (positive control); peptide H – stimulation with H peptide present in HIVconsv immunogen; vaccinated males (black) and vaccinated females (dark grey).

Both vaccine regimens elicited strong HIV-1-specific T-cell responses not detected in unvaccinated controls (Fig. 1).

### 3.2 Mortality and clinical observations

There were no unscheduled deaths during the course of the study, and no abnormal or unusual observations of clinical condition considered to be related to vaccine administration.

### 3.3 Local reactions

Reddening of the skin at the injection site was observed in 4 animals receiving DDDC and in only 1 animal in the control group (Group 1). In the 4 affected mice, reddening was observed on the day following pSG2.HIVconsv administration and persisted for up to 5 days. In 1 of these 4 animals, reddening also occurred following ChAdV63.HIVconsv administration. Following MVA.HIVconsv administration, swelling of the injection site was observed in only 1 male on day 2 and this persisted until day 6. There was no swelling noted for the rest of the animals of Groups 3 and 4. These local effects resolved fully in all animals and were considered to be related to the administration procedure rather than the vaccines.

### 3.4 Body weight

Minor changes in body weight were recorded particularly in females, but there were no statistical differences between controls and treated animals. Fluctuations in body weight do occur and overall, the changes observed in these studies reflected normal variability rather than specific association with the vaccines.

### 3.5 Food consumption

There were no apparent differences in mean food consumption between the control and treatment groups, hence no effect of vaccine administration on food consumption.

### 3.6 Ophthalmoscopy

There were no ophthalmic findings observed following treatment with any vaccine that differed from those observed pretreatment or in the control mice. Thus, there was no effect of treatment.

### 3.7 Haematology

For the DDDC regimen, significantly higher haematocrit, haemoglobin and RBC counts were observed in treated females, but not treated males when compared to controls (Table 3a). These parameters were above the concurrent control range in majority of treated animals and were associated with significantly higher MCH and MCV. Similarly, reticulocyte count was significantly higher in treated females compared to female controls. Neutrophil, lymphocyte, basophil and monocyte counts were statistically significantly elevated only in treated males. Additionally, a slight, but statistically significant elevation in platelet count was observed amongst treated males and females when compared with controls. For mainly males receiving MMM, there were statistically significant lower cell counts compared to controls in several of the parameters tested (Table 3b) but these were associated with some unusually high control values and were considered unrelated to vaccine administration. The rest of examined parameters and histopathological analysis found no evidence of vaccine-associated toxicological effects.

### 3.8 Blood chemistry

Analysis of biochemical parameters in both females and males receiving MMM (Table 3c) showed significantly higher ALT and AST levels compared to controls. For example, the mean ALT levels amongst the males were 56 and 40 U/l for treated and controls, respectively (*p* < 0.01), while the AST levels were 91 and 54, respectively (*p* < 0.01). The γ-globulin concentrations were also significantly increased in both treated sexes compared to controls, and this was reflected in the slightly higher total protein values and a marginal, but significantly lower A/G ratio. The rest of the parameters had no significant vaccine effects.

In the DDDC treatment group (Table 3c), there was a trend for higher glucose concentration in females and a higher plasma concentration of potassium in treated males and females compared to controls. The differences amongst the males reached statistical significance. In treated versus untreated females, there was a significantly lower concentration of urea, but this was considered coincidental due to overall variability. Finally, triglyceride concentration for DDDC-treated males was significantly higher than in controls while in females triglyceride concentration was lower suggesting somewhat random variations.

Overall, the differences between the vaccinated and control groups including those attaining statistical significance were only modest and considered to be within the range expected for the age and strain of animals and were, therefore, not attributed to the treatment.

### 3.9 Organ weights, macropathology and histopathology

Organ weights were adjusted for body weight as a covariate as part of the analysis. Thus, the spleens from both DDDC- and MMM-treated males and females had significantly higher weights than controls (Table 4). Histopathological examination indicated extramedullary haemopoiesis in the spleens consistent with immune stimulation. Unlike animals treated with DDDC, majority of females and males receiving MMM had enlarged popliteal and inguinal lymph nodes (Table 4). Amongst the MMM-treated both males and females, liver weights were also marginally, but significantly increased, however, there was no histopathology correlation to indicate any toxicological importance. The incidence and distribution of all other organ weights was within the expected normal range.

Microscopic examination of tissues from Groups 1 and 2 revealed histopathological changes related to DDDC vaccine administration, which was absent in control animals, only at the site of injection (Table 5), in the spleen and the right popliteal lymph node (Table 6). Thus, there was detectable interstitial and myofibre inflammation, necrosis as well as regeneration observed at the site of injection in both males and females treated with DDDC. Treatment with MMM was associated with intermyofibre, interfascicular and perimysial inflammatory cell infiltrate, which was observed in all treated animals. Dermal inflammatory cell infiltrate was also seen in majority of treated animals, but was also observed amongst the controls. Among animals treated with DDDC, increased cellularity was observed in the popliteal lymph nodes of both females and males, while plasmacytosis and perinodal inflammation were more common in males. Plasmacytosis was seen in the inguinal lymph node of one of the treated females, while another treated female showed generalised increased cellularity in the right lumbar lymph node. These lymph nodes were only examined at the microscopic level due to abnormality at macroscopic examination and because similar findings were not present in the controls or other DDDC-treated animals their significance remains unclear, however, an immune response cannot be ruled out. Increased germinal centre development was found mainly in the popliteal lymph nodes and to a lesser extent the inguinal lymph nodes of both male and female animals receiving MMM. Plasmacytosis was only seen in the popliteal lymph nodes. No other changes in the tissues presented for histopathological evaluation were considered to be related to previous treatment with the vaccines.

## 4 Discussion and conclusions

Here, we report on two GLP pre-clinical studies UNO011 and UNO012 evaluating the systemic toxicity of three candidate HIV-1 vaccines pSG2.HIVconsv DNA, ChAdV63.HIVconsv and MVA.HIVconsv administered intramuscularly, either singly or in combination, to adult male and female BALB/c mice.

In UNO011, following 3 doses of MVA.HIVconsv, no unscheduled deaths were recorded and treatment was well-tolerated and not associated with any systemic toxicological changes. The only treatment-related effects seen were those attributable to inflammatory and immune responses mainly at the injection site and in the draining immune organs, consistent with a predicted response to intramuscular vaccine administration. These changes included increased cellularity of the draining lymph nodes, high plasma γ-globulin concentration, elevated ALT/AST levels and inflammation. The MVA.HIVconsv vaccine was therefore concluded to be safe and well-tolerated in the chosen model. Based on mouse and human body weights of 25 g and 65 kg, respectively, a dose level of 2 × 10^7^ pfu given to the mouse corresponds to a dose of 5.2 × 10^10^ pfu in humans, i.e. represents a 260-fold safety margin over the clinical dose of 2 × 10^8^ pfu. The results of UNO011 supported authorisation of phase I trial HIV-CORE 001 of the MMM regimen delivering low and high doses i.m. of 5 × 10^7^ pfu and 2 × 10^8^ pfu, respectively, to HIV-1-positive individuals stable on highly active anti-retroviral treatment (EudraCT no. 2009-012662-31).

In UNO012, 3 doses of the pSG2.HIVconsv DNA followed by 1 dose of the ChAdV63.HIVconsv vaccines administered intramuscularly were also well-tolerated and not associated with any adverse systemic toxicological changes. Treatment-related effects observed were those attributable to inflammatory and immune responses at the injection site, in the draining lymph node and the spleen. Neutrophil, lymphocyte and monocyte counts were elevated in both male and female DDDC-treated animals, possibly due to an immune response to the vaccines, or secondary to the inflammatory response at the site of administration. Other findings included marginally higher red cell indices and plasma glucose in treated females, and higher platelet counts and potassium in both sexes and were also attributable to the vaccine combination, but were not considered to be adverse at the degree observed. However, a single treated female showed plasmacytosis of the right inguinal lymph node and generalised increased cellularity of the right lumbar lymph node, which were unexplained. As in UNO011, all toxicity findings in the study were consistent with the predicted response to intramuscular vaccine administration. Thus the pSG2.HIVconsv DNA and ChAdV63.HIVconsv vaccines were concluded to be safe and well-tolerated when given to BALB/c mice at dose levels of 50 μg and 5 × 10^9^ vp, respectively. Based on mouse and human body weights, a dose level of 50 μg DNA in the mouse corresponds to a dose level of 130 mg in humans. The dose level of the pSG2.HIVconsv vaccine used in the mouse study therefore represents a 32.5-fold safety margin over the proposed clinical dose of 4 mg DNA. On the same basis, a dose level of 5.95 × 10^9^ vp of ChAdV63.HIVconsv in the mouse corresponds to a dose level of 1.6 × 10^13^ vp in humans and therefore represents an approximately 3000-fold safety margin over the lower clinical dose of 5 × 10^9^ vp and an approximately 300-fold safety margin over the higher clinical dose of 5 × 10^10^ vp. The results of UNO012 and UNO011 supported authorisation of phase I trial HIV-CORE 002 in healthy, HIV-1/2-negative subjects in Oxford, UK. The trial started with a low i.m. dose of 5 × 10^9^ vp of ChAdV63.HIVconsv, followed by three groups receiving CM, DDDCM and DDDMC regimens using i.m. vaccine doses of 4 mg pSG2.HIVconsv DNA, 5 × 10^10^ vp of ChAdV63.HIVconsv and 2 × 10^8^ pfu of MVA.HIVconsv (EudraCT no. 2009-012662-31).

To support the two phase I trials above, certain tests or toxicity study features were not requested by the regulator at this stage of the vaccine platform development. These include the *N* + 1 study design, the highest doses given to humans were not given to mice, biodistribution and/or potential persistence were not tested, and coagulation parameters were not monitored. The blood volumes necessary to assess haematology, blood chemistry and clotting function cannot be abstracted from a single mouse, so unless there is an expectation of effect on clotting function, which there was not in the case of our vaccines, this is commonly excluded from regulatory mouse toxicity studies. Also biomarkers of acute inflammation are typically investigated in supplementary studies designed to address specific findings from the initial routine studies.

Over the last decade or so for investigators from the University of Oxford alone, 5, 15 and 7 similar GLP toxicity studies were carried out for DNA, recombinant MVA and ChAdV vaccines, respectively, whereby the vaccines were used alone or in combination ([9] and unpublished). Design of each study was discussed and agreed with MHRA prior to its commencement. Together, these toxicity studies supported over 96 clinical trials of over 25 experimental subunit vaccine candidates for malaria, TB, HIV, RSV, flu and cancer. For all these studies, the BALB/c mouse was chosen as the test species because of its acceptance as a predictor of toxic changes in man and was used alone. Larger animal studies were considered, but deemed not necessary. One rhesus macaque GLP study not requested by MHRA was completed [10]. Thus, for DNA-, ChAdV- and MVA-vectored vaccines, a growing body of pre-clinical and clinical data is now accumulating [9–15] facilitating safe, but rapid translation of iterative improvements in immunogen and/or regimen designs from bench to clinic.

## Figures and Tables

**Table 1:** Summary of animal treatments.

Group^a^	Treatment	Number of animals		Dosing			
		Male	Female	Day 1	Day 15	Day 29	Day 43
1	Control	10	10	PBS	PBS	PBS	PBS
2	DDDC	10	10	50 μg pSG2.HIVconsv^b^	50 μg pSG2.HIVconsv	50 μg pSG2.HIVconsv	5.95 × 10^9^ vp ChAdV63.HIVconsv^b^
3	Control	10	10	PBS	PBS	PBS	ND
4	MMM	10	10	2 × 10^7^ pfu MVA.HIVconsv^c^	2 × 10^7^ pfu MVA.HIVconsv	2 × 10^7^ pfu MVA.HIVconsv	ND

ND – not dosed.

a This summarised two studies UNO012 and UNO011 testing toxicity of DDDC and MMM regimens, respectively.

b Injected volumes i.m. of the pSG2.HIVconsv DNA and ChAdV63.HIVconsv vaccines were 50 μl.

c Injected volume i.m. of the MVA.HIVconsv vaccine was 20 μl.

**Table 2:** Tissues examined at necropsy, weighed and subjected to histopathological examination.

Macroscopic abnormalities	Parenteral sites (right hind limb, muscle and overlying skin)
Adrenals	Peyer's patches
Aorta – thoracic	Pituitary
Brain	Prostate
Caecum	Rectum
Carcass	Salivary glands
Colon	-Submandibulara,b
Duodenum	-Parotid^a^
Epididymides	-Sublinguala,b
Eyes	Sciatic nerves^a^
Femurs^a^	Seminal vesicles^b^
Gall bladder	Skeletal muscle^a^
Harderian glands	Skin with mammary glands
Heart	Spinal cord
Ileum	Spleen^b^
Jejunum	Sternum
Kidneys^b^	Stomach
Lachrymal glands	Testes^b^
Larynx	Thymus^b^
Liver^b^	Thyroid with parathyroids
Lungs^b^	Tongue
Lymph nodes	Trachea
-Mandibular	Ureters
-Popliteal	Urinary bladder
Oesophagus	Uterus and cervix^b^
Optic nerves	Vagina
Ovaries^b^	
Pancreas	

a Only one processed for examination.

b Weighed (salivary glands weighed together, lung weight included mainstem bronchi).

**Table 3a:** Haematology for DDDC.^a^

Group/sex^b^		Hct (l/l)	Hb (g/dl)	RBC (×10^12^/l)	Retic (%)	MCH (pg)	MCHC (g/dl)	MCV (fl)	WBC (×10^9^/l)	N (×10^9^/l)	L (×10^9^/l)	E (×10^9^/l)	B (×10^9^/l)	M (×10^9^/l)	LUC (×10^9^/l)	Plt (×10^9^/l)
1M	Mean	0.518	16.0	10.69	2.21	15.0	30.8	48.4	4.48	0.87	3.37	0.12	0.01	0.09	0.03	1100
	SD	0.0073	0.25	0.159	0.115	0.15	0.17	0.49	1.667	0.295	1.297	0.041	0.005	0.033	0.026	48.5
2M	Mean^c^	0.512	15.9	10.67	2.28	14.9	31.0	48.1	6.80**	1.20*	5.28**	0.13	0.01*	0.13*	0.05	1198**
	SD	0.0097	0.23	0.210	0.144	0.31	0.42	0.80	1.172	0.223	1.108	0.051	0.005	0.042	0.012	65.0
1F	Mean	0.492	15.3	9.87	2.26	15.5	31.2	49.8	2.89	0.54	2.21	0.07	0.00	0.05	0.02	1018
	SD	0.0090	0.33	0.217	0.344	0.14	0.46	0.83	1.522	0.332	1.181	0.033	0.005	0.021	0.013	23.2
2F	Mean^c^	0.509*	16.0*	10.46**	2.88*	15.3*	31.4	48.7**	4.00	0.85	2.94	0.09	0.01	0.09	0.02	1119**
	SD	0.0167	0.64	0.387	0.683	0.17	0.68	0.86	2.407	0.545	1.796	0.053	0.005	0.057	0.021	90.3

Hct – haematocrit; Hb – haemoglobin; RBC – erythrocyte; Retic – reticulocyte; MCHC – mean cell haemoglobin concentration; MCV – mean cell volume; WBC – total white cell count; N – neutrophils; L – lymphocytes; E – eosinophils; B – basophils; M – monocytes; LUC – large unstained cells; Plt – platelet.

a Animals received 3 doses i.m. of pSG2.HIVcosv DNA followed by 1 dose i.m. of ChAdV63.HIVconsv.

b 1M – males control; 2M – males receiving DDDC; 1F – females control; 2F – females receiving DDDC.

c *t*-Test: *p < 0.05, **p < 0.01.

**Table 3b:** Haematology for MMM.^a^

Group/sex^b^		Hct (l/l)	Hb (g/dl)	RBC (×10^12^/l)	Retic (%)	MCH (pg)	MCHC (g/dl)	MCV (fl)	WBC (×10^9^/l)	N (×10^9^/l)	L (×10^9^/l)	E (×10^9^/l)	B (×10^9^/l)	M (×10^9^/l)	LUC (×10^9^/l)	Plt (×10^9^/l)
1M	Mean	0.466	15.8	10.62	2.59	14.8	33.8	43.9	1112	6.90	0.98	5.61	0.10	0.01	0.15	0.05
	SD	0.0143	0.43	0.318	0.192	0.21	0.48	0.57	23.9	0.866	0.259	0.683	0.015	0.006	0.048	0.010
2M	Mean^c^	0.458	15.3*	10.58	2.49	14.5**	33.4	43.3*	1053**	4.25**	0.66**	3.28**	0.17**	0.00**	0.10*	0.02**
	SD	0.0078	0.40	0.241	0.242	0.14	0.52	0.53	41.0	0.937	0.079	0.849	0.049	0.005	0.044	0.008
1F	Mean	0.449	15.3	10.08	2.50	15.2	34.1	44.6	973	3.93	0.73	2.99	0.09	0.00	0.11	0.02
	SD	0.0059	0.39	0.218	0.277	0.20	0.59	0.54	49.8	1.246	0.219	1.097	0.034	0.005	0.031	0.011
2F	Mean^c^	0.463*	15.6	10.28	2.30	15.2	33.7	45.0	1047	4.31	0.75	3.33	0.12	0.01*	0.08	0.03
	SD	0.0172	0.38	0.322	0.389	0.43	1.09	0.35	90.8	1.476	0.317	1.242	0.031	0.004	0.016	0.019

Hct – haematocrit; Hb – haemoglobin; RBC – erythrocyte; Retic – reticulocyte; MCHC – mean cell haemoglobin concentration; MCV – mean cell volume; WBC – total white cell count; N – neutrophils; L – lymphocytes; E – eosinophils; B – basophils; M – monocytes; LUC – large unstained cells; Plt – platelet.

a Animals received 3 doses i.m. of MVA.HIVcosv.

b 1M – males control; 2M – males receiving MMM; 1F – females control; 2F – females receiving MMM.

c *t*-Test: *p < 0.05, **p < 0.01.

**Table 3c:** Blood Chemistries–Parameters with statistically significant differences.

Group^a^ Sex		ALT (U/l)	AST (U/l)	Bili (μmol/l)	Urea (mmol/l)	Creat (μmol/l)	Gluc (mmol/l)	Trig (mmol/l)	Na (mmol/l)	K (mmol/l)	Ca (mmol/l)	Phos (mmol/l)	Total Prot (g/l)	Alb (g/l)	Gamma (g/l)	A/G ratio
MMM^b^ (*n* = 10 per group)																
1M	Mean	40	54		6.16			1.00					52		1	1.62
	SD	9.3	10.0		0.287			0.241					1.4		0.0	0.119
2M	Mean	56**	91**		7.01*			1.56**					54**		2**	1.50*
	SD	12.9	30.8		1.002			0.519					1.3		0.4	0.079
1F	Mean	40	74						151	4.1	2.48		51	33	1	1.97
	SD	19.8	32.5						1.1	0.36	0.052		1.6	1.6	0.0	0.157
2F	Mean	57*	117**						152*	3.7*	2.56*		55**	35*	3**	1.73**
	SD	16.4	33.5						1.4	0.38	0.463		1.6	1.1	0.5	0.062

DDDC^c^ (*n* = 10 per group)																
1M	Mean					10		1.52		4.0			49		30	1.48
	SD					1.3		0.233		0.31			1.2		0.8	0.076
2M	Mean					8*		2.09*		4.3*			48*		28**	1.42*
	SD					1.5		0.768		0.34			1.6		1.0	0.065
1F	Mean			1	8.70		10.12	1.49			2.38	2.31				
	SD			0.0	1.276		1.256	0.624			0.059	0.322				
2F	Mean			2**	6.72**		12.43*	0.91*			2.46**	3.03**				
	SD			0.5	1.028		2.257	0.222			0.066	0.571				

ALT – alanine aminotransferase; AST – aspartate aminotransferase; Bili – Total bilirubin; Creat – creatinine; Gluc – glucose; Trig – triglycerides; Phos – inorganic phosphorus; Alb – Albumin; Gamma – γ-globulin; A/G – albumin/globulin ratio.

a 1M – males control; 2M – males receiving MMM; 1F–females control; 2F–females receiving MMM.

b Animals received 3 doses i.m. of MVA.HIVcosv.

c Animals received 3 doses i.m. of pSG2.HIVcosv DNA followed by 1 dose i.m. of ChAdV63.HIVconsv. ^d^*t*-Test: *p < 0.05; **p < 0.01.

**Table 4:** Tissue enlargement.

Group	Treatment	Day of examination	Number of animals/tissues examined	Organ/tissue examined^a^					
				Spleen		Popliteal lymph node (right)		Inguinal lymph node (right)	
				Male	Female	Male	Female	Male	Female
1	Control	Day 50	10	0	0	0	0	0	0
2	DDDC^b^	Day 50	10	0	5/10	0	0	0	0
3	Control	Day 36	10	0	0	0	0	0	0
4	MMM^c^	Day 36	10	0	0	9/10	8/10	6/10	8/10

a The numbers in the table indicate the number of animals in each group with significantly enlarged organs.

b Animals received 3 doses i.m. of pSG2.HIVcosv DNA followed by 1 dose i.m. of ChAdV63.HIVconsv.

c Animals received 3 doses i.m. of MVA.HIVconsv.

**Table 5:** Microscopic pathology at the injection site.^a^

Observation	Group 1 (control)		Group 2 (DDDC^b^)		Group 3 (control)		Group 4 (MMM^c^)	
	Male	Female	Male	Female	Male	Female	Male	Female
Myofibre inflammation	0	0	10/10	9/10	–	–	–	–
Myofibre regeneration	0	0	5/10	8/10	–	–	–	–
Myofibre necrosis/degeneration	0	0	3/10	3/10	–	–	–	–
Interstitial inflammation	0	0	4/10	9/10	–	–	–	–
Intermyofibre/interfascicular/perimysial inflammatory cell infiltrate	–	–	–	–	0	0	10/10	10/10
Dermal inflammatory cell infiltrate	–	–	–	–	4/10	5/10	6/10	8/10
Number of tissues examined	10	10	10	10	10	10	10	10

**Table 6:** Microscopic changes in the lymph nodes.^a^

Observation	Group 1 (control)		Group 2 (DDDC^b^)		Group 3 (control)		Group 4 (MMM^c^)									
	Popliteal		Inguinal		Popliteal		Inguinal		Popliteal		Inguinal		Popliteal		Inguinal	
	M	F	M	F	M	F	M	F	M	F	M	F	M	F	M	F
Increased cellularity – generalised	0	1/10	0	0	7/10	6/9	0	0	–	–	–	–	–	–	–	–
Plasmacytosis	0	0	0	0	4/10	1/9	0	1/10	0	0	–	–	4/10	4/10	–	–
Perinodal Inflammation	0	0	0	0	5/10	1/9	0	0	–	–	–	–	–	–	–	–
Increased germinal centre development	–	–	–	–	–	–	–	–	0	0	–	–	9/10	10/10	5/6	7/8
Number of tissues examined	8	10	10	10	10	9	10	10	9	9	0	0	10	10	6	8

a The numbers in the table indicate the number of animals in each group with significant microscopic changes.

b Animals received 3 doses i.m. of pSG2-HIVcosv DNA followed by 1 dose i.m. of ChAdV63.HIVconsv.

c Animals received 3 doses i.m. of MVA.HIVconsv.
